# Administration of Δ9‐Tetrahydrocannabinol (THC) Post‐Staphylococcal Enterotoxin B Exposure Protects Mice From Acute Respiratory Distress Syndrome and Toxicity

**DOI:** 10.3389/fphar.2020.00893

**Published:** 2020-06-16

**Authors:** Amira Mohammed, Hasan Alghetaa, Muthanna Sultan, Narendra P. Singh, Prakash Nagarkatti, Mitzi Nagarkatti

**Affiliations:** Department of Pathology, Microbiology and Immunology, School of Medicine, University of South Carolina, Columbia, SC, United States

**Keywords:** acute respiratory distress syndrome, Δ9-Tetrahydrocannabinol (THC), Staphylococcal enterotoxin B (SEB), CB2 receptor, miR-34a, microRNA, Myeloid-Derived Suppressor Cells, Tregs

## Abstract

Acute Respiratory Distress Syndrome (ARDS) is a life-threatening complication that can ensue following *Staphylococcus aureus* infection. The enterotoxin produced by these bacteria (SEB) acts as a superantigen thereby activating a large proportion of T cells leading to cytokine storm and severe lung injury. Δ9Tetrahydrocannabinol (THC), a psychoactive ingredient found in Cannabis sativa, has been shown to act as a potent anti-inflammatory agent. In the current study, we investigated the effect of THC treatment on SEB-induced ARDS in mice. While exposure to SEB resulted in acute mortality, treatment with THC led to 100% survival of mice. THC treatment significantly suppressed the inflammatory cytokines, IFN-γ and TNF-α. Additionally, THC elevated the induction of regulatory T cells (Tregs) and their associated cytokines, IL-10 and TGF-β. Moreover, THC caused induction of Myeloid-Derived Suppressor Cells (MDSCs). THC acted through CB2 receptor as pharmacological inhibitor of CB2 receptors blocked the anti-inflammatory effects. THC-treated mice showed significant alterations in the expression of miRNA (miRs) in the lung-infiltrated mononuclear cells (MNCs). Specifically, THC caused downregulation of let7a-5p which targeted SOCS1 and downregulation of miR-34-5p which caused increased expression of FoxP3, NOS1, and CSF1R. Together, these data suggested that THC-mediated alterations in miR expression in the lungs may play a critical role in the induction of immunosuppressive Tregs and MDSCs as well as suppression of cytokine storm leading to attenuation of SEB-mediated lung injury.

## Introduction

Acute lung injury (ALI) and its more severe form, the acute respiratory distress syndrome (ARDS) are caused by a myriad of etiologies such as toxic inhalation, lung contusion, pancreatitis, pneumonia, sepsis, and trauma (Frutos-Vivar et al., 2006). Incidence of ARDS in USA is 78.9 per 100,000 persons/year and the mortality rate is 38.5% (Rubenfeld et al., 2005; Rubenfeld and Herridge, 2007). The ARDS incidence increases with age, being more robust and serious in older people (Rubenfeld et al., 2005). ARDS is also triggered by *Staphylococcus aureus* and its enterotoxin, SEB. ARDS is a life-threatening complication of infection especially caused by methicillin-resistant *Staphylococcus aureus* (CA-MRSA). SEB acts as a super-antigen by activating a large proportion of T cells expressing certain Vβ-specific T cell receptors. Such an activation leads to release of massive amounts of cytokines (Mason et al., 1998) and consequent injury to various organs, including the lungs. ARDS remains difficult to treat and thus far, no pharmacological protocol is effective in modifying the course of this clinical disorder leading to significant morbidity and mortality (Rubenfeld and Herridge, 2007). Thus, understanding the mechanisms and developing new treatment modalities against ARDS and cytokine storm is timely and critical.

Delta-9-Tetrahydrocannabinol (THC) is one of at least 113 cannabinoids identified in cannabis plant. THC is the principal psychoactive constituent of cannabis. THC acts as a ligand for two receptors, CB1 and CB2. While its psychoactive properties are attributed to its ability to activate CB1 expressed in the CNS, because CB2 receptors are primarily expressed on immune cells, THC also exerts significant immunosuppressive activity. There is growing evidence suggesting that THC can suppress inflammation though activation of CB2 through multiple pathways. These include: a) Switch from Th1 to Th2 (Yuan et al., 2002), b) differentiation of Tregs (Hegde et al., 2008), and c) induction of Myeloid-derived Suppressor Cells (MDSCs) (Sido et al., 2015b). d) Induction of apoptosis in activated T cells and dendritic cells (McKallip et al., 2002; Rieder et al., 2010).

In recent years, microRNAs (miRs) have been focus of many studies in the regulation of genes expression (Friedman et al., 2009). miRs are a class of small non-coding RNAs roughly 20–22 nucleotides in length with extensive secondary structure (Lee et al., 1993). miRs regulate gene expression by either directly binding to a specific sequences located at 3′UTR regions of the genes or degrading messenger RNAs (Ipsaro and Joshua-Tor, 2015). Several studies from our lab have shown a role for miRs in the regulation of inflammatory genes in various diseases models (Alghetaa et al., 2018; Alharris et al., 2018; Al-Ghezi et al., 2019b; Neamah et al., 2019). In a previous study, we noted that THC treatment attenuated SEB-mediated lung injury and that it was associated with downregulation of miRNA-18a, which targeted Pten (phosphatase and tensin homolog), an inhibitor of the PI3K/Akt signaling pathway (Rao et al., 2015).

In the current study, we investigated if THC would induce anti-inflammatory Tregs and MDSCs in mice exposed to SEB and if their induction was regulated by miR. Our data demonstrated that THC decreased the expression of two key miRs, let7a-5p and miR-34-5p which targeted the expression of several signaling molecules that targeted the induction or functions of Tregs and MDSCs.

## Materials and Methods

### Mice

Female adult C3H/HeJ mice were purchased from The Jackson laboratory. All the mice were housed in a pathogen-free conditions animal facility of University of South Carolina School of Medicine. The facility is accredited by AALAC. All experiments using mice were performed under protocols approved by the Institutional Animal Care and Use Committee (IACUC) of USC.

### Chemicals and Regents

Staphylococcus enterotoxin B (SEB) was purchased from Toxin Technologies (Sarasota, FL, USA). THC was procured from (NIH National Institute on Drug Abuse, National Institutes of Health, Bethesda, MD, USA). The following reagents (culture medium reagents: RPMI 1640, L-Glutamine, Penicillin-Streptomycin, HEPES, FBS, and PBS) were purchased from Invitrogen Life Technologies (Carlsbad, CA). We purchased the following antibodies: Phycoerythrin (PE)-conjugated anti-CD4 (clone: GK 1.5), BV510-conjugated anti-Gr-1 (RB6-8c5), Alexa floura700-conjugated anti-CD11b (clone: M1/70), Alexa Flour 488-conjugated anti-Foxp3 (clone MF-14), and Foxp3 Fix/Perm buffer from Biolegend (San Diego, CA, USA). Fc Block reagent was purchased from BD Pharmingen (Carlsbad, CA). True-Nuclear™ Transcription Factor Buffer Set was purchased from Biolegend. RNeasy and miRNAeasy Mini kits, miScript primer assays kit, and miScript SYBR Green PCR kit were purchased from QIAGEN (QIAGEN, Valencia, CA). iScript and miScript cDNA synthesis kits were purchased from Bio-Rad (Madison, WI) and Epicentre’s PCR premix F and Platinum *Taq* DNA Polymerase kits were purchased from Invitrogen Life Technologies (Carlsbad, CA).

CB2 receptor antagonist SR144528 (SR144) was purchased from TOCRIS (Minneapolis, MN, USA) and was reconstituted and stored according to manufacturer’s recommendations. ELISA kits for IFN-γ, TNF-α, M-CSF, IL-10, and TGF-β (ELISA MAX™ Standard SET Mouse) were purchased from Biolegend.

### Exposure of Mice With SEB and THC Treatments

SEB was delivered as a “Dual Dose” as described previously (Huzella et al., 2009). This approach causes 100% mortality with low concentrations of SEB and triggers ARDS in C3H/HeJ mice. In brief, SEB dissolved in sterile PBS was administered first by the intranasal (i.n.) route at a concentration of 5 μg per mouse in 25 μl volume. Two hours later, a second dose of SEB was delivered (i.p.) at a concentration of 2 μg per mouse in a 100 μl volume. THC was given in three doses: First dose of vehicle or THC was given at 20 mg/kg, i.p. immediately after first SEB exposure. Second and third doses of THC were given i.p. at 10 mg/kg after 24 and 48 h after the first dose of THC. THC dose used is relevant to pharmacological use in humans to treat cancer patients to prevent nausea and develop appetite. For example, THC dose of 20 mg/kg in mice translates to 60 mg/m^2^, based on body surface area normalization, and in patients, THC has been recommended for a maximum dose of 90 mg/m^2^/day, as also described in our previous work (Rao et al., 2015). SEB‐exposed mice displayed signs of lethargy, hunching, ruffled fur and respiratory distress and such mice were euthanized. For survival studies, mice that were administered with SEB + Veh or SEB + THC, as described above, were observed for 40 days and any mice showing distress were euthanized. To study the immune response and miR expression, mice were euthanized 72 h after SEB + Veh or SEB + THC exposure and lung mononuclear cells (MNCs) and Bronchoalveolar Lavage Fluid (BALF) were collected as previously described (Elliott et al., 2016).

### Evaluation of Lung Functions

To understand the effect of SEB and THC treatments, lung functions were measured using whole-body plethysmography (Buxco, Troy, NY, USA). Each mouse was restrained in a two-chamber plethysmographic tube and was allowed to acclimatize as previously described (Elliott et al., 2016). The lung function was calculated as the Specific Airway Resistance (sRaw), Specific Airway Conductance (sGaw), delay time (dT), and Peak expiratory flow (PEF).

### Analysis of Lung Infiltrated MNCs

We analyzed lung infiltrated MNCs post-SEB exposure and THC treatments. In brief, mice were euthanized 72 h post-SEB exposure. The lungs from SEB + vehicle and SEB + THC groups were first perfused with heparin-containing PBS, then harvested and homogenized using Stomacher^®^ 80 Biomaster blenders from Seward (Davie, FL, USA) in 10 ml of sterile PBS. Cells, after washing with cold PBS twice, were layered carefully onto Ficoll-Histopaque^®^-1077 (Sigma-Aldrich, St Louis, MO, USA) at a 1:1 ratio. MNCs were separated by density gradient centrifugation as described previously (Rieder et al., 2012) and enumerated by Trypan blue exclusion. To determine the phenotypical characteristics of the infiltrating cells, MNCs were stained with the fluorescent conjugated antibodies Phycoerthyrin (PE)-conjugated anti-CD4 (clone: GK 1.5), (BV510)-conjugated anti-Gr-1 (RB6-8c5), (Alexa floura700)-conjugated anti-CD11b (clone: M1/70) from Biolegend (San Diego, CA, USA). Intracellular staining of Foxp3 was carried out using Biolegend’s Foxp3 Fix/Perm buffer set following manufacturer’s instructions and using anti-foxp3 Alexa floura 488 (clone MF-14) from Biolegend. The isotype controls were used as negative controls. The stained cells were analyzed using BD Celesta flow Cytometry and DIVA software. In addition, we also performed histopathology of lungs by using H&E staining as described (Rao et al., 2015).

### Analysis of Cytokines in BALF

To assess cytokines post-SEB exposure and treatment with vehicle or THC, we analyzed BALF (Broncho Alveolar Lavage Fluid) from lungs of mice. BALF was obtained by binding the trachea with a suture and excising the lung along with the trachea as described previously (Rieder et al., 2012). In brief, sterile, ice-cold PBS was injected through the trachea to aspirate the fluid. The collected BALF was centrifuged to obtain the supernatants containing cytokines. ELISA was performed using ELISA MAX™ standard kits from Biolegend and following the protocol of the company to determine cytokines present in BALF.

### Quantitative Real-Time (Q-PCR) to Determine the Expression of FOXP3, IL10, NOS2, and TGF-βR3

We performed Q-PCR to determine the expression of FOXP3, IL10, NOS2, and TGF-βR3 in lung infiltrated MNCs. To this end, cDNAs were generated using total RNAs isolated from mice treated with SEB + Veh or SEB + THC. We used SSO Advanced™ SYBR green PCR kit from Bio-Rad (Hercules, CA, USA). The following primers for FOXP3, IL10, NOS2, and TGF-β were used. The following primers were used:

FoxP3 primers:
  Forward primer: 5′-CCCATCCCCAGGAGTCTTG-3′
  Reverse Primer: 5′-ACCATGACTAGGGGCACTGTA-3′
IL-10 Primers:
  Forward Primer: 5′-GCTCTTACTGACTGGCATGAG-3′
  Reverse Primer: 5′-CGCAGCTCTAGGAGCATGTG-3′
NOS2 primers:
  Forward Primer: 5′-TTCAGATCCCGAAACGCTACAC-3′
  Reverse Primer: 5′-ACAATCCACAACTCGCTCCAAG-3′
TGF- βR2 Primers:
  Forward Primer: 5′-GGAGAAGTGAAGGATTACGAGC-3′
  Reverse Primer: 5′-CACACGATCTGGATGCCC-3
GAPDH Primers
  Forward Primer: 5′-AGGTCGGTGTGAACGGATTTG-3′
  Reverse Primer: 5′-TGTAGACCATGTAGTTGAGGTCA-3′

The following PCR cycles (40 cycles) conditions: 15 min at 95°C (initial activation step), 15 s at 94°C (denaturing temperature), 30 s at 60°C (annealing temperature), and 30 s at 70°C (extension temperature and fluorescence data collection) were used. Expression of mRNAs was calculated using normalized expression 2^−ΔΔ*CT*^, where CT is the threshold cycle to detect fluorescence. Fold change of mRNA levels was normalized to GAPDH (a house keeping gene).

### AmiR Arrays Analyses to Evaluate MicroRNAs Profile in Lung Infiltrated MNCs

Total RNAs including miRs were isolated from lung infiltrated MNCs harvested from mice exposed to SEB and treated with vehicle or THC using miRNAeasy kit from Qiagen and following the protocol of the company (Qiagen). Using Affymetrix miR array (GCS3000 System, version 4), miRs arrays were performed. Expression (fold change) profile of over 3,000 miRs were obtained from the raw array data and only those miRs that showed more than two-fold change were considered for further analysis. The selected miRs were further analyzed for their targets and alignments using TargetScan, microRNA.org, and miRWalk and their database, as described (Tomar et al., 2015; Alghetaa et al., 2018; Kadhim et al., 2018). Furthermore, selected miRs were analyzed for their role in various diseases and pathways using ingenuity pathway analysis (IPA) software. miRs from various groups were also analyzed for their relationship using Lucid Chart or Venn diagram.

### Transcriptome Arrays to Analyze Gene Expression in Lung MNCs

To understand SEB-induced regulation of genes following treatment with THC in lung infiltrated MNCs, we performed to Transcriptome arrays using GCS3000 Affymtrix System. In brief, total RNA from purified lung MNCs was isolated using miRAeasy Mini kit and following the protocol of the company (Qiagen). In brief, single strand cDNAs were synthesized in a thermal cycler incubating the reaction mix (total RNAs and cDNA reagents) for 60 min at 25°C, 60 min at 42°C, and then at 4°C. Next, second strand cDNAs were synthesized from first-stranded cDNAs to generate double-stranded cDNAs. Complementary RNAs (cRNAs) were synthesized according to Eberwine method (Van Gelder et al., 1990) using T7 RNA polymerase to *in vitro* transcription (IVT) of second-stranded cDNA into cRNA by incubating the IVT master mix with second-stranded cDNA for 16 h at 40°C. Magnetic microbeads (Affymetrix, USA) were used to purify the cRNAs. Purified cRNAs (15 µg) was used to synthesize second cycle of ss-cDNA by the reverse transcription of cRNA using 2^nd^-cycle primers. The remaining cRNAs were removed using RNase H. Microbeads-purified ss-cDNA was fragmented by uracil-DNA glycosylase and using purinic/pyrimidinic endonuclease 1 at the unnatural dUTP residues to break the DNA strand. The fragmented ss-cDNAs were then labeled using labeling master mix just before the hybridization process. Hybridization master mix (contains fragmented and labeled ss-cDNAs) was first loaded into ClariomD chip (Affymetrix, USA) and then the GeneChip was incubated in Hybridization Oven 645 (Affymetrix, USA) for 16 h at 45°C with rotation at speed of 60 rpm. Next, GeneChip Hybridization Wash and Stain kit (Affymetrix, USA) was used to wash and stain the chips containing hybridized ss-cDNA at RT by using GeneChip Fluidics Station 450 (Affymetrix, USA) for about 2 h. Once the staining and washings were done, the chips were scanned using GeneChip Scanner (Affymetrix, USA) and the obtained raw data was analyzed using Transcriptome analysis console (TAC) to determine the expression of genes in MNCs.

### Real-Time (qRT-PCR) to Validate the Expression of Select miRs and Associated Genes

To validate the expression of select miRs (let-7a-5p and miR-34a-5p) and associated genes (FoxP3, SOCS1, NOS1, and CSF1R) in lung infiltrating MNCs post exposure to SEB and treated with vehicle or THC, qRT-PCR assay was performed. In brief, total RNA including miR from lung infiltrating MNCs was isolated using miRNAeasy kit from Qiagen and following the manufacturer’s instructions. miScript primer assay kit and miScript SYBR Green PCR kit from QIAGEN were used and qRT-PCR assay was performed following the protocol of the company (QIAGEN, Valencia, CA). For qRT-PCR, 40 cycles using the following conditions: 15 min at 95°C (initial activation step), 15 s at 94°C (denaturing temperature), 30 s at 60°C (annealing temperature), and 30 s at 70°C (extension temperature and fluorescence data collection) were used. The data were normalized to miRs against internal control miR (SNORD96A) and fold change of miRs was calculated against control miR (SNORD96A). To determine the expression of genes, mRNA levels were normalized to GAPDH (a house keeping gene) (Al-Ghezi et al., 2019a; Sarkar et al., 2019). Predicted miRNA‐target gene alignments were determined with online miRNA database (www.microrna.org). The details of primers used for miRs (let-7a-5p and miR-34a-5p) and associated genes (Foxp3, SOCS1, NOS1, and CSF1R) are described below:

CSF1R Primers
  Forward Primer: 5′-CTTCAGCATCTTCACAGCCACCTT-3′
  Reverse Primer: 5′-AGAGCTATGAGGGCAACAGTT-3′
NOS1 Primers
  Forward Primer: 5′-AAAACACCCTTGTTACCACAC-3′
  Reverse Primer: 5′-AGCTCTTGTCCGTACCAC-3′
SOCS1 Primers
  Forward Primer: 5′-ACAAGCTGCTACAACCAGGG-3′
  Reverse Primer: 5′-ACTTCTGGCTGGAGACCTCA-3′

### Transfection of Splenocytes With miR-34a

Splenocytes harvested from naïve C3H/HeJ mice were cultured in complete RPMI medium supplemented with 10% FBS and 1% penicillin/streptomycin. The cells were seeded at density of 2 × 10^5^ cells/well in a 24-well plate and were activated with 1 µg/ml SEB overnight. The following day, SEB-activated cells were transfected using Qiagen HiPerfect Transfection Reagent and 20nM of miR-34a mimic (syn-mmu-miR-34-5p-mimic: 5′-UGGCAGUGUCUUAGCUGGUUGU-3′) or anti-mmu-miR-34-5p-inhibitor (5′-UGGCAG UGUCUUAGCUGGUUGU-3′) or transfection reagent alone (mock) as previously described (Busbee et al., 2015). The transfected cells were cultured for 48 h. The transfected cells were then collected and used for total RNAs including miRs isolation. Total RNA was then used for validation of miR-34a and Foxp3, NOS1, and CSF1R expression.

### Statistical Analysis

All statistical analyses were carried out using GraphPad Prism v6 Software (San Diego, CA, USA). In all the experiments, the number of mice used was 4–5 per group, unless otherwise specified. Student’s t-test was used to compare the two groups, whereas multiple comparisons were made using one-way ANOVA, followed by *post hoc* analysis using Tukey’s method. p < 0.05 was considered statistically significant. Individual *in vitro* experiments were performed in triplicate. Each experiment was performed independently at least three times to test the reproducibility of results. Survival analysis was carried out using a log-rank.

## Results

### THC Treatment of Mice Exposed to Staphylococcal Enterotoxin-B (SEB) Protects Them From ARDS

**Figure 1:**
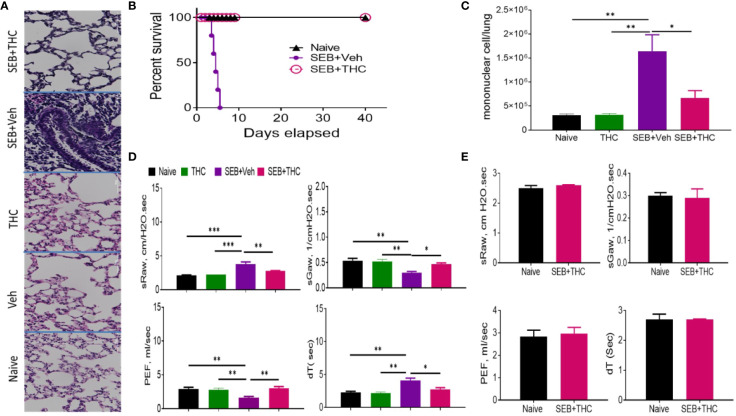
THC attenuates SEB-induced ARDS in mice. SEB was administered first by the intranasal (i.n.) route followed by a second dose of SEB was delivered (i.p.) as described in Methods. Mice were then treated with vehicle (SEB + Veh) or THC (SEB + THC). **(A)** Representative images of histopathological H and E staining sections of excised lung tissues from naïve, Veh, THC, SEB + Veh, and SEB + THC **(B)** Showing survival of mice 40 days post lethal dual-dose exposure to SEB and treated with THC or Vehicle and naive group. **(C)** Total number of MNCs isolated from the lungs. **(D)** Pulmonary performance (sRaw, specific resistance airway; sGaw, specific conductance airway; PEF, peak of expiratory flow; dT, delayed time of translocation of air from thoracic to nasal canal and verse versa). **(E)** Evaluation of mice by Whole-body non-invasive plethysmograph Buxco 72 h post-SEB-induced ALI. Pulmonary performance as shown in panel **(D)**, was evaluated in mice that survived after SEB + THC for 40 days when compared to naïve mice. In all these experiments, groups of five mice were used and data confirmed in three independents experiments. Statistical significance is depicted as *p < 0.05, **p < 0.01, ***p < 0.001 between the groups.

Histopathological evaluation using H&E staining of the lungs post‐SEB + Vehicle exposure demonstrated that the lung architecture was significantly damaged when compared to lungs from naïve or vehicle-treated mice, with excessive cellular infiltration in the alveolar and interstitial spaces of the tissue (**Figure 1A**). In contrast, SEB + THC group had attenuated lung injury as evidenced by decreased cell infiltration and damage to lung architecture. The lungs from mice treated with THC alone showed no significant changes when compared to naïve or vehicle-treated mice. The mice exposed to dual dose of SEB and treated with vehicle (SEB + Veh) succumbed to death within 72–120 h post-exposure, but 100% of SEB-exposed mice treated with THC (SEB + THC) survived (**Figure 1B**), consistent with our previous studies (Rao et al., 2015). Enumeration of MNCs infiltrating the lungs showed that SEB + Veh group had significant increase in such cells when compared to naïve, or THC alone group, while SEB + THC group showed significant reduction in MNCs (**Figure1C**). Upon analysis of lung functions using plethysmography, SEB + Veh exposure caused a significant increase in the sRaw and dT due to the obstruction in the airway, while this effect was reversed in mice treated with SEB + THC (**Figure 1D**). Moreover, SEB + Veh–treated mice recorded a significant decrease in sGaw and PEF, while THC treatment led to reversal of these effects (**Figure 1D**). All pulmonary functions listed above were also evaluated 40 days post-SEB exposure in the survival group (SEB + THC) and compared with naïve mice of the same age. The results of pulmonary functions showed that the survival group had normal pulmonary functional similar to naïve mice (**Figure 1E**).

### THC Treatment Suppresses Inflammatory Cytokines IFN-γ and TNF-α but Promotes the Generation of Anti-Inflammatory Cytokines TGF-β and IL-10 in BALF of Mice

**Figure 2:**
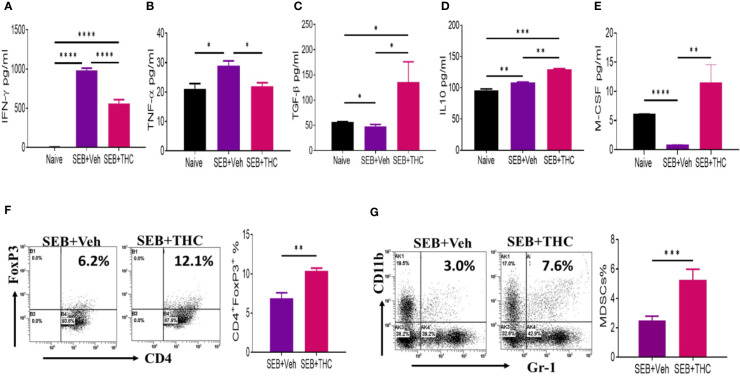
THC suppresses inflammatory cytokines and induces Tregs and MDSCs. Mice were treated with SEB + Veh or SEB + THC as described in **Figure 1** legend, and 72 h later the assays were performed. Panels **(A–E)** depict various cytokines detected in BALF using ELISA. **(F)** Lung MNCs stained for CD4+/FoxP3+ Tregs showing a representative experiment using flow cytometry. **(G)** Staining lung MNCs for expression of CD11b+ and Gr-1+ MDSCs, showing a representative experiment using flow cytometry. Vertical bars show data from groups of five mice with Mean ± SEM. Data was confirmed in three independent experiments. Statistical significance is depicted as *p < 0.05, **p < 0.01, ***p < 0.001, ****p < 0.0001 between the groups.

SEB, being a superantigen, is known to cause massive release of cytokines. Therefore, we examined the generation of key cytokines (IFN-γ, TNF-α, TGF-β, IL-10, and M-CSF) in BALF isolated from mice that were exposed to SEB + Veh or SEB + THC. The data showed that inflammatory cytokines, IFN-γ and TNF-α were upregulated in mice exposed to SEB + Veh, when compared to naïve mice (**Figures 2A, B**), but significantly decreased in mice treated with SEB + THC (**Figures 2A, B**). Upon analysis of anti-inflammatory cytokines (TGF-β and IL-10), we noted significant decrease in both TGF-β and IL-10 in BALF of SEB + Veh–exposed mice, when compared to naïve mice (**Figures 2C, E**). However, THC treatment significantly increased the levels of TGF-β and IL-10 in BALF of SEB-treated mice (**Figures 2C, E**). We also noted that SEB + Veh–treated group had significantly lower levels of M-CSF while treatment with THC caused marked increase in M-CSF. Next, we examined the presence of various immune cell subsets in the lung infiltrating MNCs. To this end, we stained MNCs with various fluorescein-conjugated anti-mouse antibodies to detect regulatory T cells (Treg: CD4+/FoxP3+) and Myeloid Derived Suppressor Cells (MDSCs: CD11b+/Gr-1+ cells) and analyzed using a Flow Cytometer. These data showed that mice exposed to SEB + THC had significantly more percentages of Tregs (**Figure 2F**) and MDSCs (**Figure 2G**) in lung infiltrating MNCs, when compared to SEB + Veh group (**Figures 2F, G**). Data taken together suggested that THC treatment promoted the generation of anti-inflammatory cytokines (TGF-β and IL-10) and immunosuppressive cells (Treg and MDSCs) while significantly suppressing the generation of inflammatory cytokines (IFN-γ and TNF-α).

### THC-Mediated Induction of Treg and MDSCs Is CB2-Dependent

**Figure 3:**
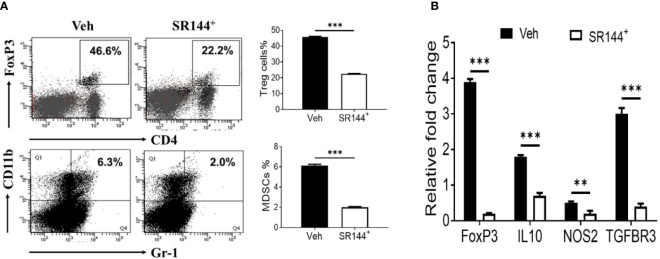
THC promotes the generation of Tregs and MDSCs through CB2 receptor activation. Spleen cells were cultured with SEB + THC in the presence of vehicle or CB2 antagonist, SR-144. **(A)** shows *in vitro* generation of Tregs and MDSCs in the presence of vehicle or CB2 antagonist (SR144). Vertical bars show data from four mice with Mean ± SEM. **(B)** Expression of genes (FoxP3, IL-10, NOS2, TGF-βR3) using RT-PCR in SEB + THC-activated splenocytes cultured in the presence of vehicle or CB2 antagonist (SR144). Statistical significances are depicted as **p < 0.01, ***p < 0.001 between the groups.

Because immune cells express CB2 receptors (Yang et al., 2015), we next investigated if THC was acting through CB2. To that end, splenocytes were first activated with SEB and then treated with CB2 antagonist (SR144528) at a concentration of 10 μm or Vehicle, 1 h before treating the cells with THC. We observed that the presence of CB2 antagonist caused decreased THC-mediated induction of Tregs and MDSCs when compared to the controls (**Figure 3A**). Furthermore, upon examination of expression of several associated genes (FoxP3, IL-10, NOS1, and TGF-β R3) by performing qRT-PCR, we observed significant decrease in their expression in cells treated with CB2 antagonist, when compared to Vehicle controls (**Figure 3B**). Data taken together demonstrated that THC acts through CB2 receptor in modulating immune cell functions.

### THC Treatment Altered miR Profile in Lung Infiltrated MNCs

**Figure 4:**
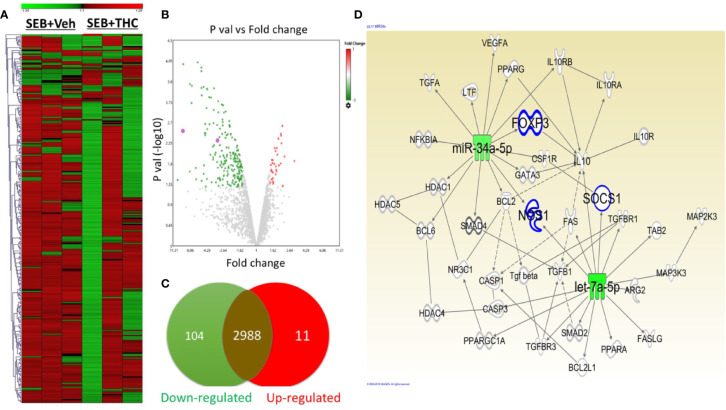
THC alters miR expression in lung infiltrated MNCs exposed to SEB. Mice were treated with SEB + Veh or SEB + THC as described in **Figure 1** legend. MNCs from the lungs were isolated and screened for miR expression as described in Methods. **(A)** Heat map of all miRs in SEB + Veh and SEB + THC groups. **(B)** Volcano plot depicts statistically dysregulated miRNAs in SEB + THC vs. SEB + Veh. **(C)** Venn diagram of miRs with two-fold change (upregulated, downregulated or no change). **(D)** Shows pathways and relationship between miRs and genes that regulate Treg, MDSC induction, proliferation, and signaling post-IPA analysis of dysregulated miRs.

Recent studies from our lab have shown that THC caused dysregulation of miR expression in mice (Mehrpouya-Bahrami et al., 2019) (Al-Ghezi et al., 2019b). To investigate the effect of SEB and THC on miR profile and correlate with Treg and MDSCs induction and suppression of SEB-induce ARDS in mice, we performed miRs arrays using total RNAs including miRs from lung infiltrated MNCs from mice exposed to SEB and treated with vehicle or THC. There were more than 3,103 of miRs that were analyzed by arrays (**Figure 4A**). However, there were only 115 miRs out of 3,103 miRs that were dysregulated greater than two-fold in SEB + THC group when compared to SEB + Veh–treated MNCs (**Figure 4A**). The volcano plot shows the p value and significant fold change in miRNAs (**Figure 4B**). Additionally, Venn diagram (**Figure 4C**) shows that there were (2,988) miRs that showed no change, whereas, 11 miRs were upregulated and 104 miRs were downregulated. Furthermore, upon analysis of 115 dysregulated miRs using IPA software for genes related to molecules that regulate Treg, MDSC induction, proliferation and signaling, we observed a direct relationship between various miRs and the target genes including miR-34a-5p and the target genes FoxP3, IL-10, NOS1, CSF1Rr, and SOCS1, and miR-let-7a-5p that targets SOCS1 and IFN-γ genes (**Figure 4D**). These data demonstrated that THC-mediated alterations in miRs may regulate the expression of various genes (FoxP3, IL-10, NOS1, CSF1Rr, SOCS1, and IFN-γ, etc.), which in turn may regulate generation of Treg and MDSCs.

### Effect of THC on Regulation of Genes to Identify Mechanistic Pathways That Trigger SEB-Induced ARDS in Mice

**Figure 5:**
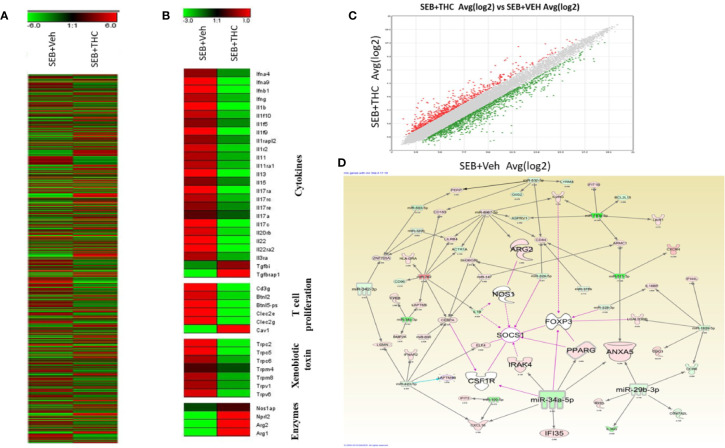
THC treatment affects transcriptional profiles of gene expression in lung infiltrated MNCs of mice exposed to SEB. Groups of five mice were treated with SEB + Veh or SEB + THC as described in **Figure 1** legend. MNCs from the lungs were isolated and subjected to transcriptional analysis as described in Methods. **(A)** Heat map of all genes in SEB + Veh and SEB + THC groups. **(B)** Heat map showing specific genes associated with cytokines, T cells proliferation, xenobiotic toxin, and in enzymes in SEB + Veh vs. SEB + THC groups. **(C)** Scatter plot up- and downregulated genes post calculation of total genes (linear). **(D)** Shows relationship between genes and miRs post IPA analysis of dysregulated genes detecting the genes regulating Tregs and MDSCs to miRs.

Next, we examined the altered expression of genes in lung infiltrated MNCs by performing transcriptome arrays using total RNA isolated from MNCs post-exposure to SEB and treated with vehicle or THC as described in detail in *Materials and Methods*. Data obtained from Transcriptome arrays showed dysregulation of a large number of genes in MNCs of the two groups (**Figure 5A**). **Figure 5A** shows Heat map of the dysregulated genes in SEB + Veh and SEB + THC groups. Based on the two-fold change in the gene expression, Heat map showed dysregulation of many genes related to cytokines, T cell proliferation, Xenobiotic toxin, and enzymes in SEB + Veh and SEB + THC groups (**Figure 5B**). Scatter plot analysis also showed dysregulation of a large number of genes (**Figure 5C**). Upon analysis of dysregulated genes in the two groups using IPA Analysis software, we observed a strong relationship between miR array and transcriptome array data obtained from MNCs. We observed that miR-34a showed relationship with FoxP3, CSF1R, IRAK4, SOCS1, NOS1, IF135, and ANXA5 (**Figure 5D**).

### Validation of Selective miRs Expression and Their Target Genes in MNCs

**Figure 6:**
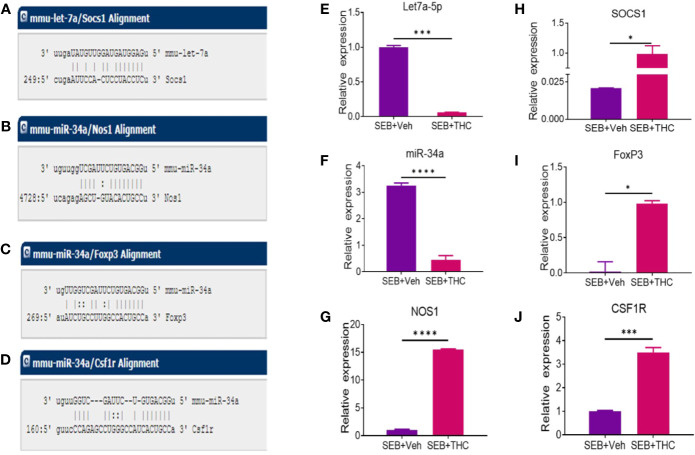
Validation of selective miRs and targeted genes: Groups of five mice were treated with SEB + Veh or SEB + THC as described in **Figure 1** legend. MNCs from the lungs were isolated and screened for miR expression and inflammatory markers by quantitative RT-PCR. **(A)** Showing binding affinity between miR-let-7a-5p and SOCS1. **(B–D)** Showing binding affinity between miR-34a-5p and NOS1, FoxP3 and CSF1R. (**E**, **F**) Expression of miR-let-7a-5p and miR-34a-5p. **(G–J)** Expression of targeted genes NOS1, SOCS1, FoxP3 **(I)**, and CSF1R **(J)** in lung infiltrated MNCs. Gene expression in panels **(E–G)** was calculated as relative gene expression (ΔC_T_). Statistical significances are depicted as *p < 0.05, ***p < 0.001, ****p < 0.0001 between the compared groups.

We selected two miRs (miR-let-7a-5p and miR-34a-5p) based on the complementary binding affinity of miR-let-7a-5p with SOCS1 gene (**Figure 6A**) and miR-34a-5p with NOS1 (**Figure 6B**), FoxP3 (**Figure 6C**), and CSF1R (**Figure 6D**). We performed qRT-PCR using mouse miR-let-7a-5p and miR-34a-5p-specific sets of forward and reverse primers as described in *Materials and Methods*. Data obtained from qRT-PCR showed significant downregulation of both miR-let-7a-5p (**Figure 6E**) and miR34a-5p (**Figure 6F**) in MNCs of SEB + THC group, when compared to SEB + Veh group. Next, we also examined the expression of miR-let-7a-5p target gene NOS1 and miR-34a-5p target genes (SOCS1, FOxP3, and CSF1R) by performing qRT-PCR as described in *Materials and Methods*. There was significant upregulation all these four genes in MNCs of SEB + THC group, when compared to SEB + Veh group (**Figure 6G**, NOS1; H, SOCS1; I, FoxP3; and J, CSF1R). These data suggested that THC alters the expression of miRs in MNCs which may regulate induction and functions of Tregs, MDSCs and cytokine production.

### Analysis of miR-34a-5p Expression and That of Its Targeted Genes NOS1, FoxP3, and CSF1R

**Figure 7:**
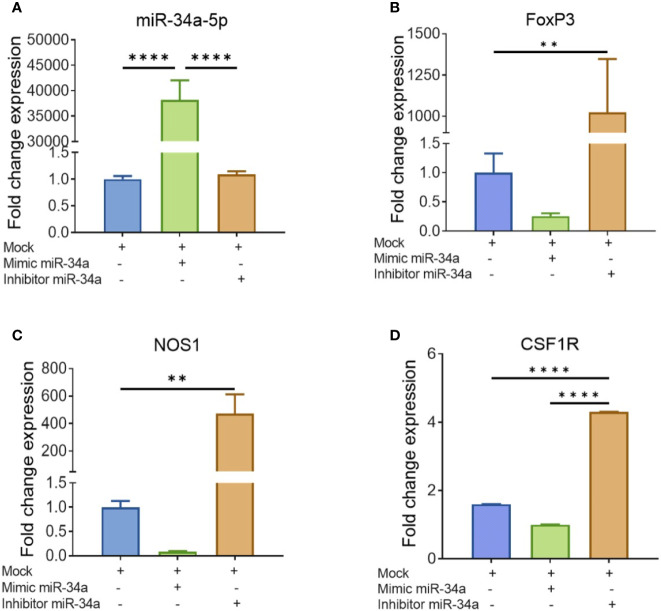
Validation of genes targeted by miR-34a-5p: Splenocytes harvested from a group of four naïve C3H/HeJ mice were cultured and activated with SEB overnight followed by transfection with mock, mimic or inhibitor of miR-34a-5p. RT-PCR was used to detect the levels of targeted genes. **(A)** miR-34a-5p expression with mock, mimic or miR-34-5p inhibitor. **(B–D)** Expression of FoxP3 **(B)**, NOS1 **(C)**, and CSF1R **(D)** in the presence of mock, mimic or miR-34-5p inhibitor. Data presented as vertical bars represent Mean ± SEM of triplicates. Different gene expression in panels **(A–D)** was calculated as normalized to mock control group gene expression (ΔΔC_T_). Statistical significances are depicted as **p < 0.01, ****p < 0.0001 between the compared groups.

To further corroborate the role of miRs, we performed transfection assays to determine if miR34a-150-5p targeted the expression of NOS1, FoxP3, SOCS1, and CSF1R. In brief, splenocytes were cultured overnight in the presence of SEB and the following day, the cells were Mock-transfected or transfected with mature miR-34a-5p or anti-miR-34a-5p inhibitor. The transfected cells were cultured for 48 h and the expression of miR-34a-5p (**Figure 7A**) and NOS1, FoxP3, and CSF1R (**Figures 7B, D**) genes was determined by performing qRT-PCR. The splenocytes transfected with mature miR-34a-5p showed significantly upregulated expression in these cells (**Figure 7A**). However, transfection of splenocytes with anti-miR-34a-5p inhibitor showed downregulated expression of miR-34a-5p when compared to cells transfected with miR-34a-5p mimic (**Figure 7A**). To further understand the role of miR-34a-5p, we performed qRT-PCR to determine the expression of NOS1, FoxP3, and CSF1R genes in the transfected splenocytes (**Figures 7B, D**). The expression of NOS1, FoxP3, and CSF1R was significantly suppressed in the transfected splenocytes in the presence of miR-34a-5p mimic (**Figures 7B, D**). However, the expression of the above genes was significantly increased in the splenocytes in the presence of anti-miR-34a-5p inhibitor (**Figures 7B, D**). Data obtained from this study showed that miR-34a-5p is directly involved in the regulation of NOS1, FoxP3, and CSF1R.

## Discussion

Staphylococcus enterotoxin B (SEB) is produced by gram-positive bacteria, *Staphylococcus aureus*, which is ubiquitous that adversely affects about one-third of the general population worldwide. It is responsible for staphylococcal food poisoning in humans and has been produced by some countries as a biological weapon (Wu et al., 2019). SEB acts as a super antigen as it activates a large number of T cells such as those expressing Vβ8, triggering cytokine storm, acute toxic shock, and multi-organ failure leading to mortality (Krakauer et al., 2016; Zhang et al., 2017). SEB is also known to cause ARDS. Previous studies from our lab have shown that dual dose administration of minute doses of SEB into C3H/HeJ mice caused severe lung injury and mortality through the expansion of T lymphocytes and massive release of pro-inflammatory cytokines such as IFN-γ, while treatment with THC rescued these mice from SEB-mediated toxicity (Rao et al., 2015). While it is difficult to treat SEB-mediated toxicity, elegant studies demonstrated that short peptide mimetics of a conserved superantigen domain that activates T cells can block cytokines and prevent SEB-induced mortality in mice (Arad et al., 2000). Additionally, SEB must bind directly into the dimer interface of CD28, and also its coligand B7-2, to trigger T-cell hyperactivation. Thus, preventing access of SEB to CD28 or B7-2 has also been shown to block lethality (Arad et al., 2011; Levy et al., 2016).

In the previous study, we injected two doses of THC (20 mg/kg) prior to SEB injection and one dose after SEB exposure (Rao et al., 2015). In the current study, we tested to see if treatment with THC after exposure to SEB was also effective and we noted that it was indeed able to reverse the toxicity of SEB thereby providing translational significance. While in the previous study, we noted that SEB induced miRNA-18a, which targeted Pten (phosphatase and tensin homolog), an inhibitor of the PI3K/Akt signaling pathway, thereby suppressing the induction of Tregs, THC treatment inhibited miRNA-18a thereby reversing the effects of SEB (Rao et al., 2015). Because THC is known to trigger multiple pathways to suppress inflammation, we investigated additional mechanisms involving miRs that may target immunosuppressive cells such as MDSCs. Our results showed that THC induced both MDSCs and Tregs and that their induction correlated with downregulation of miR-let-71-5p and miR-34a-5p, which targeted key molecules such as NOS1, FoxP3, CSF1R, and SOCS1.

In the current study, we observed that THC suppressed SEB-induced inflammation and improved the pulmonary functions of the lung which was associated with activation and recruitment of Tregs and MDSCs to the site of inflammation (**Figure 1A**). One of the drawbacks of using THC in patients with ARDS is that it is psychoactive because of its ability to activate CB1 receptors in the brain. Interestingly, in the current study, we noted that THC-mediated effect on ARDS was regulated, at least in part, through CB2 receptor, inasmuch as blocking CB2 with a CB2 antagonist (SR 144) decreased the ability of THC to induce MDSCs and Treg as well anti-inflammatory cytokines (**Figures 3A, B**). Because CB2 select agonists do not exhibit psychoactive properties, they may be better suited to treat ARDS in patients. However, in-depth studies on their use *in vivo* need further evaluation. THC has been shown to mediate potent anti-inflammatory properties through multiple pathways which include recruitment of MDSCs and regulatory T cells (Nagarkatti et al., 2009; Hegde et al., 2010; Sido et al., 2015a). Previous studies from our lab showed that the anti-inflammatory properties of THC was mediated at least in part through induction of MDSCs and a significant increase in GCSF which recruits MDSCs (Hegde et al., 2008). The role of cannabinoid receptor involvement in our ARDS model, is consistent with the observation that CB1 and CB2 receptors are expressed in the lungs and the bronchial tissue (Turcotte et al., 2016). Also, endocannabinoids may play a role in suppressing inflammation during ARDS as suggested by the observation that increases in endocannabinoid, 2-Arachidonoylglycerol, triggers anti-inflammatory effects in a murine model of LPS-induced ARDS (Costola-de-Souza et al., 2013).

Several studies from our lab and others have characterized MDSCs as potent immunosuppressive cells (Gabrilovich et al., 2001; Gabrilovich and Nagaraj, 2009) MDSCs may also be induced at sites of inflammation and may prevent tissue injury by downregulating inflammatory T cell responses (Bronte, 2009). Cauley et al. showed that repeated systemic administration of staphylococcal enterotoxin A (SEB) led to induction of tolerance *via* accumulation of MDSCs in the spleen (Cauley et al., 2000). We have recently also shown that THC attenuated chronic colitis in IL-10 knockout mice through MDSCs induction (Singh et al., 2012). Rieder et al. demonstrated that THC-induced immunosuppressive MDSCs play a critical role in suppressing lung-injury (Rieder et al., 2012). There are studies that have shown the role of MDSCs in the suppression of pro-inflammatory cytokines and other mediators such as G-CSF, GM-CSF, IL-1β, IL-12, and IFN-γ (Gabrilovich and Nagaraj, 2009).

Similar to MDSCs, the role of Tregs in suppressing inflammation has been well established. In this study, we observed that THC promoted generation of Tregs (**Figure 2F**) and associated cytokines such as IL-10 (**Figure 2D**) and TGF-β (**Figure 2C**) in lung infiltrating MNCs. These data are consistent with previous studies showing that THC-mediated induction of Tregs attenuates ConA-induced liver injury in mice (Hegde et al., 2008). In another study involving colitis, it was shown that activation of CB2 by JTE907 promotes the differentiation of Th0 cells into the Treg cell phenotype, which was characterized by the expression of FoxP3, TGF-β, and IL-10 (Gentili et al., 2019). Tregs are known to mediate their immunosuppressive functions through release of anti-inflammatory cytokines, such as IL-10 and TGF-β (Huang et al., 2006). In this study, we also observed upregulated expression of IL-10 in lung infiltrating MNCs (**Figure 2D**). It should be noted that chronic inflammatory reactions induced by a variety of stimuli can trigger fibrosis involving scarring of tissues which is attributed to excess deposition of extracellular matrix components including collagen (Wynn, 2008). Studies in both humans and animal models strongly suggest that TGF-β plays a pivotal role in the pathogenesis of pulmonary fibrosis (Zhang et al., 2019). Thus, while in acute inflammatory reactions, TGF-β may play immunosuppressive role, during chronic inflammation leading to fibrosis of lungs, TGF-β may play a negative role. However, because our model involves acute inflammation, it is likely that TGF-β induced by THC may play a protective role in preventing ARDS.

Many studies have been shown that miRs play a major role in the regulation of gene expression and the immune responses (Arora et al., 2017), including autoimmunity and inflammation (Singh et al., 2015). We and others have shown previously that miRs play a critical role in promoting anti-inflammatory functions and immune suppression (Singh et al., 2015) (Zhou et al., 2014) (Singh et al., 2016) (Kadhim et al., 2018) (Guan et al., 2016). In the current study, therefore, we investigated if treatment with THC following SEB injection would alter the expression of miRs in lung immune cells. In this study, we observed that THC caused altered expression of a large number of miRs in lung infiltrating MNCs of mice exposed to SEB, when compared to mice treated with vehicle (**Figure 4D**). Upon pathway analysis of miRs using IPA, we identified two downregulated miRs (miR-let-71-5p and miR-34a-5p) in MNCs that may play significant role in THC-induced immune suppression. MiR-let-7a-5p showed strong binding affinity with complementary sequences of 3′UTR regions of SOCS1 gene whereas miR-34a-5p showed strong binding affinity with NOS1, FoxP3, and CSF1R genes. Because THC downregulated the expression of miR-let-7a-5p and miR-34a-5p, these data suggested that this effect was responsible for increased expression of SOCS1, NOS1, FoxP3, and CAF1R in the MNCs. The role of miR-34a-5p to regulate the expression of NOS1, FoxP3, and CSF1R was further confirmed by performing transfection experiments and qRT-PCR (**Figure 7**). There was significant downregulation of NOS1, FoxP3, and CSF1R expression in splenocytes that were transfected with miR-34a-5p mimic while the expression of NOS1, FoxP3, and CSF1R was significantly upregulated in splenic cells that were transfected with anti-miR-34a-5p inhibitor. NOS1 is a neuronal isozyme known to promote inflammation. However, NOS1 can metabolize arginine to produce nitric oxide. Arginine is an essential metabolite for T cells and thus, NOS1 can deprive T cells of arginine thereby suppressing proinflammatory T cells (Rodriguez et al., 2017). In fact, MDSCs use such a mechanism involving direct deprivation of arginine. MDSCs have been shown to express CSFR1 (Lee et al., 2019), also known as macrophage colony-stimulating factor receptor (M-CSFR). In addition, activation of CSFR1 is involved in the differentiation of immunosuppressive macrophages (Bonelli et al., 2018).

FoxP3 is a master regulator of the development and functions of Tregs (Lu et al., 2017). Our data that FoxP3 is induced by THC in SEB model is consistent with previously published studies in models such as Graft-vs-Host disease and experimental model of Multiple Sclerosis (Pandey et al., 2011; Al-Ghezi et al., 2019b). In the previous study involving treatment with THC prior to SEB injection, we found that THC downregulated miRNA-18a, which targeted Pten, an inhibitor of the PI3K/Akt signaling pathway, thereby inducing T-regulatory cells (Rao et al., 2015). In the current study involving post-SEB treatment with THC, we identified yet another miR (miR-34a-5p) that may target FoxP3 directly and thereby lead to induction of Tregs. SOCS1 is a negative regulator of cytokine signaling. Thus, it suppresses the induction of inflammatory cytokines. In addition, SOCS1 maintains the stability of Tregs and prevents their plasticity to differentiate into inflammatory Th17 and Th1 cells when exposed to inflammatory cytokines (Ilangumaran et al., 2017). Thus, the induction of NOS1, FoxP3, CSF1R, and SOCS1 by THC following downregulation of miR-34a-5p and miR-let-7a-5p, may lead to activation of a wide array of immunosuppressive pathways involving cytokines, Tregs, MDSCs, and macrophages.

In summary, the current study suggests that treatment of mice with THC post-SEB challenge protects mice from SEB-mediated toxicity by inhibiting inflammation and ARDS through the modulation of miRs. Because SEB is a super antigen that drives cytokine storm, our studies suggest that THC is a potent anti-inflammatory agent that has the potential to be used as a therapeutic modality to treat SEB-induced ARDS. This study also suggests that THC may mediate its effects through downregulating the expression of miR-let-7a-5p and miR-34a-5p that target the expression of SOCS1, NOS1, FoxP3, and CSF1R and consequently trigger immunosuppressive MDSCs and Treg as well as directly suppress inflammatory cytokines, leading to attenuation of SEB-induced ARDS in mice.

It is of interest to note that a significant proportion of Coronavirus disease 2019 (COVID-19) patients come down with sepsis and ARDS accompanied by cytokine storm. Because currently there is no effective treatment against ARDS, a significant percentage of such COVID-19 patients die from severe damage to the lungs and other organs, caused by cytokine storm (Mehta et al., 2020). SEB being a super antigen, also triggers cytokine storm and lung injury as seen from the current study, however, clearly additional studies are needed to investigate if the mechanisms involved are similar and whether cannabinoids can be used to treat COVID-19 related ARDS.

## Data Availability Statement

The raw data support the conclusions of this article are available at NCBI.nlm.nih.gov/geo under accession number GSE148649.

## Ethics Statement

The animal study was reviewed and approved by Institutional Animal Care and Use Committee (IACUC) of University of South Carolina.

## Author Contributions

AM, PN, and MN designed all the experiments. AM performed all experiments under supervision of PN and MN. AM collected and analyzed data and wrote the manuscript. HA, MS, and NS contributed to analyze data. HA contributed to microarray analysis. NS revised first copy of the manuscript and which was approved by all authors. PN and MN supervised the work from designing to finalizing the manuscript for journal submission.

## Funding

This work was financially supported with NIH grants P01AT003961, R01AI123947, R01AI129788, and P20GM103641 to PN and MN, and Iraqi Ministry of High Education and Scientific Research (MoHESR) fellowship for AM.

## Conflict of Interest

The authors declare that the research was conducted in the absence of any commercial or financial relationships that could be construed as a potential conflict of interest.

## Abbreviations

ALI, acute lung injury; ARDS, acute respiratory distress syndrome; BALF, broncho-alveolar lavage fluid; CB2, cannabinoid receptor2; CSF1R, colony stimulating factor-1 receptor; FoxP3, forkhead box P3; IFN-γ, interferon gamma; IL-10, interleikin-10; M-CSF, monocyte-colony stimulating factor; MDSCs, myeloid derived suppressor cells; miR, micro-RNA; MNCs, mono-nuclear cells, NOS1, nitric oxide synthase-1; Pten, Phosphatase and tensin; SEB, staphylococcal enterotoxin-B; SOCS1, suppressor of cytokine signaling-1; TGF-β, transforming growth factor-beta, THC, Δ9-Tetrahydrocannabinol, TNF-α, tumor necrosis factor-alpha; Treg, T regulatory cells; UTR, untranslated region.
